# Stretchable, Flexible, Scalable Smart Skin Sensors for Robotic Position and Force Estimation

**DOI:** 10.3390/s18040953

**Published:** 2018-03-23

**Authors:** John O’Neill, Jason Lu, Rodney Dockter, Timothy Kowalewski

**Affiliations:** Department of Mechanical Engineering, University of Minnesota, 111 Church St SE, Minneapolis, MN 55401, USA; luxxx544@umn.edu (J.L.); dockt036@umn.edu (R.D.); timk@umn.edu (T.K.)

**Keywords:** skin, stretchable, robotics, collaborative control

## Abstract

The design and validation of a continuously stretchable and flexible skin sensor for collaborative robotic applications is outlined. The skin consists of a PDMS skin doped with Carbon Nanotubes and the addition of conductive fabric, connected by only five wires to a simple microcontroller. The accuracy is characterized in position as well as force, and the skin is also tested under uniaxial stretch. There are also two examples of practical implementations in collaborative robotic applications. The stationary position estimate has an RMSE of 7.02 mm, and the sensor error stays within 2.5±1.5 mm even under stretch. The skin consistently provides an emergency stop command at only 0.5 N of force and is shown to maintain a collaboration force of 10 N in a collaborative control experiment.

## 1. Introduction

As robotic systems have proliferated in the home, industrial, and medical areas, robots have worked in progressively closer proximity to the humans around them. In order to safely coexist, sensors have been utilized to allow robots to avoid dangerous interactions, with varying success [1]. This field is in need of a modular, durable and affordable sensor to provide a sense of touch that can easily be applied to any robot geometry and can stretch and flex to conform to the geometry without losing the ability to continuously measure the position and magnitude of a touch like human skin [2].

The skin could allow robots to intuitively learn to control the torque that they are applying [3], or to learn their inverse dynamics models without a priori knowledge [4]. The sensing skin could also give robots an improved situational awareness, allow them to interact in a more human-like manner through nonverbal touch, and collaborate more intuitively on complicated tasks in constrained environments. For example, to complete a robotic surgery surgeons and nurses communicate only verbally; however, a tactile sensing skin could allow the robot and surgeon to sense the need for space by another person in the operating room.

However, the design of a robotic skin is a difficult task [5]. One possible solution to humans and robots working in close proximity is the ‘soft’ joints of the Baxter Robot (Rethink Robotics, Boston, MA, USA) where springs are placed between the motors and joints, allowing them to absorb any collision with a human [6] at the expense of increased complexity in hardware and software. Other methods from the ROBOSKIN project include using depth sensing or computer vision to track bodies around the robot for collision avoidance [7]. Vision-based methods can be combined with tactile sensors [8] and can even be used to calibrate tactile sensors [9], and enable advanced detection of contact shapes [10]. However, the vision portion is limited to line-of-sight applications and does not allow intuitive, nonverbal communication by a human pushing on a robot without a tactile sensor that can stand on its own.

One method of developing a large skin sensor is to create an array of force sensors, known as tactile pixels (taxels), which provide knowledge of whether a touch has occurred at that point. In order to provide flexibility, some skins have utilized a pliable media upon which rigid taxels are mounted, such as a flexible printed circuit board or polydimethylsiloxane (PDMS) silicone. Force sensors include standard strain gauges [11], capacitive sensors [12], piezoresistive force sensors [13], and polyvinylidene fluoride (PVDF) force sensors [14]. However, due to the use of rigid sensors, the resulting devices are not continuously flexible or stretchable, although some flexibility can be added to rigid sensor arrays by placing PDMS above a rigid array [15]. Further, the sensor array’s position resolution is limited to the size of the taxels themselves, leading to a trade-off between resolution and complexity due to the need to reference each taxel individually. Even if the sensors are intelligently accessed through modular systems [16], the number of electrodes increases linearly (2n), thereby increasing the sensor’s area (n×n).

In order to improve upon the rigid arrays of sensors, other groups have created flexible sensor pads, for example a tactile sensor designed by Choi et al. based on 0.5 mm${}^{2}$ PVDF sensor arrays which are embedded in polyester film [17]. The small size of the PVDF sensors allow flexibility that surpasses that of the larger sensor arrays; however, they still require a large number of electrodes, which complicates their installation and use. A flexible tactile sensor comprised of a polyester sheet skin embedded with a grid of silver-polymer conductive traces has been developed by Papakostas et al. [18]. Although the grid contacts create a piezoresisitive force sensor and the conductive traces can be made flexible, the sensor still results in $n^{2}$ electrodes for an n×n sensor array. A PDMS interlocked micro-structure skin by Park et al. [19] additionally provides an estimate of the shear forces present by measuring resistance. A PDMS-based sensor developed by Tomo et al. uses magnetic hall-effect sensors to detect force with a deformable sensor [20] which can be embedded into robotic components already using PDMS [21]. A system developed for the SAPHARI project by Cirillo et al. uses photodetectors to observe deformations of a deformable silicone layer [22] which can then be used to manage unintentional collisions as well as be used as human-machine interface [23]. Another system developed by Back et al. uses an array of deformable taxels which are monitored by optical fibers [24] to detect force, requiring one optical fiber per taxel.

Conductive liquids have allowed a novel approach for stretchable and flexible sensors. For example, the use of microchannels containing eutectic gallium-indium within a PDMS substrate [25] can be arrayed to cover larger areas [26]. This provides skins that are continuously stretchable, unlike the discretely stretchable skins created by the aforementioned methods; however, the channels correspond to discrete locations, creating a non-continuous collection of sensing pads. A continuously flexible and stretchable skin could be applied to robot arms such as the Kuka LBR (KUKA Roboter GmbH, Augsburg, Germany) or the Baxter, conforming to the arbitrary geometries of the robot and providing continuous sensing without dead spots. Stretchability is required if the skins are manufactured in mass, as it avoids the need to create custom three-dimensional sensors for each robot geometry encountered.

A different method of position estimation is to use electrical impedance tomography. This involves putting a large number of electrodes around the perimeter of the skin and detecting a change in impedance when the path between two electrodes would pass through the point of contact. For example Pugach et al. have shown that conductive rubber sheets can be manufactured by adding carbon into the material prior to curing [27], and Lee et al. have shown that such sheets can be used for a multi-directional strain mapping [28]. However this method is limited by the requirement that the object touching the skin be electrically conductive, which may not be the case in human-robot interaction. Further, the scalability of this method is poor as spatial and temporal resolution are not maintained when the size of the area is increased. The number of electrodes located around the perimeter and their corresponding wires scale by 4n for $n^{2}$ discrete taxels. Finally, the limits of stretchability for this method are unclear, leaving uncertainty as to whether the method could actually be applied to arbitrary robot links.

An interesting method that is fully stretchable is that shown by Lacasse et al. involving a silicone with carbon black (CB) combined with a conductive fabric [29]. This material is cut into strips to create a weave of discrete taxels in a similar manner to that of earlier sensors, and the resistance in each sensor is used to determine a touch. This sensor is truly stretchable; however, it still suffers from the issue of discrete taxels leading to increased wires or decreased resolution, discussed above. Another concern with this method is that the visco-elastic properties of silicone may interfere with the sensing modality and require dynamic calibration to account for it, which would further limit the ability to customize the skin.

It has been shown that carbon nanotubes (CNT) exhibit a piezoresistive effect when formed into a film [30] a foam [31] or a yarn [32]. This method however suffers from hysteresis, and does not inherently provide position information [33].

To date, there exists no continuously stretchable and flexible skin sensor that provides (1) continuous position sensing without dead spots; (2) a small number of wires that does not increase with size or accuracy; (3) easy manufacturability with low cost electronics and processing requirements; and (4) the ability to adapt to the wide range of robot geometries available. We propose a solution that provides a low-cost, continuously flexible and stretchable smart skin sensor. We exploit a two-dimensional potentiometer effect by using a PDMS elastomer sheet doped with carbon nanotubes (CNT). This work builds on preliminary work done by Walz et al. to develop a tissue-tracking two-dimensional potentiometer for medical training [34], and the addition of a conductive fabric top layer by Lu et al. [35]. In this paper, we characterize the accuracy, flexibility, and limitations of this smart skin sensor and provide two examples of practical implementations in collaborative robotic applications.

An early concept of this work was introduced in [36], where we presented the linear position method and a cubic force fit, as well as emergency stop and evasive action experiments. This paper additionally provides an alternative and independent diagonal position method, as well as an improved neural network model for estimating force including differentiation between low and high forces. Importantly, this paper also evaluates the quality of the positional sensing over stretches up to 133% and the piezoresistive effects of stretch on bulk resistance, investigating and substantiating the claim of skin stretchability that was unsubstantiated in the original work. Finally, this paper shows a more detailed, confirmatory analysis of the microstructure of the skin and uses Akaike Information Criterion to rigorously evaluate the polynomial fit order.

## 2. Methods

The smart skin development consists of four primary components: the physical sensor, the electronics and algorithms, an optional offline finite element simulation, and the calibration routine. These components are outlined in the following section.

### 2.1. Sensor Design

**Figure 1:**
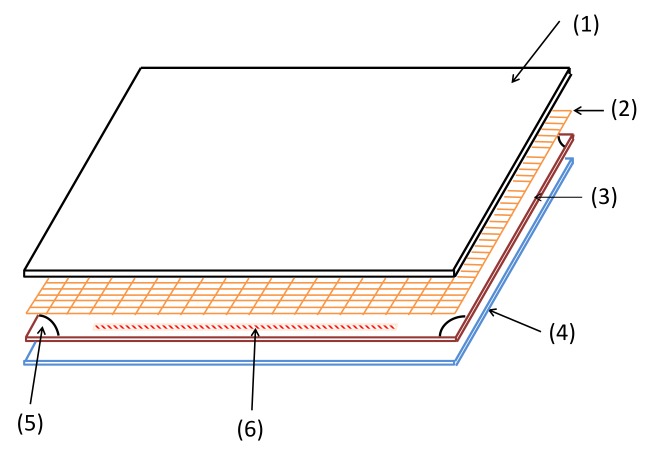
Smart skin exploded view with layers: (1) Ag. Nylon (2) Perforated Layer (3) PDMS-CNT Layer (≈ 50 μm thick) (4) PDMS Substrate (1.5 mm thick) (5) Stretchable Contact (Ag Nylon + CNT-PDMS) (6) Stretchable Adhesive.

**Figure 2:**
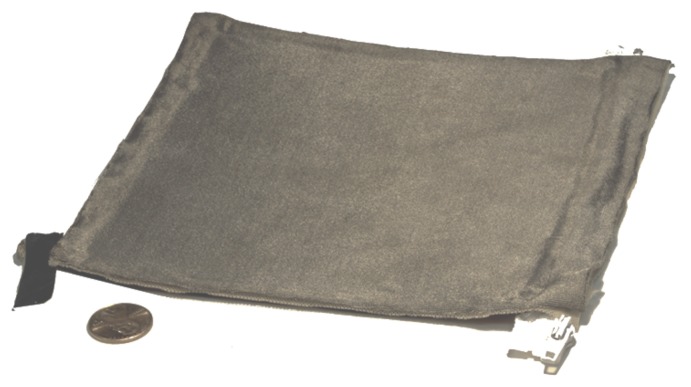
The assembled smart skin with bonded zippers for easier attachment.

**Figure 3:**
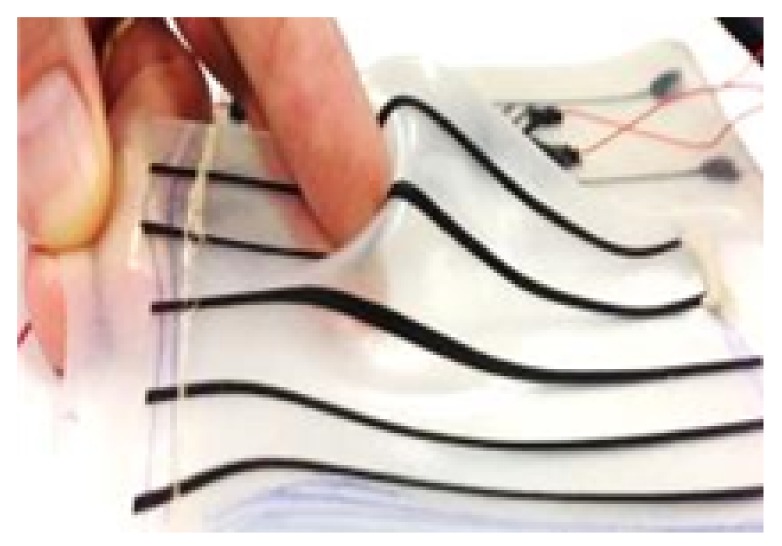
A close-up of the PDMS substrate with CNT stripes to indicate stretch.

The proposed smart skin sensor consists of three stretchable, flexible layers as shown in Figure 1, and fully assembled in Figure 2. The upper layer is a nylon fabric made conductive by a silver coating (Medtex, Statex Productions, Bremen, Germany). The intermediate layer is a perforated cloth (Powermesh Fabric, 99% polyester, 1% spandex) that provides a non-conductive intermediate layer. The lower layer, shown in detail in Figure 3, consists of a PDMS substrate that is approximately 1.75 mm thick. This layer also has a 100 μm uniformly bonded CNT-PDMS coating (7-SIGMA Inc., Minneapolis, MN, USA) which is oriented towards the intermediate layer. All layers are cut to a square of the desired pad dimensions, which is 14.7 cm square for the robot interaction experiments and 10.0 cm for the stretch experiments. The layers are bonded around the edges using a non-conductive adhesive (7-SIGMA).

**Figure 4:**
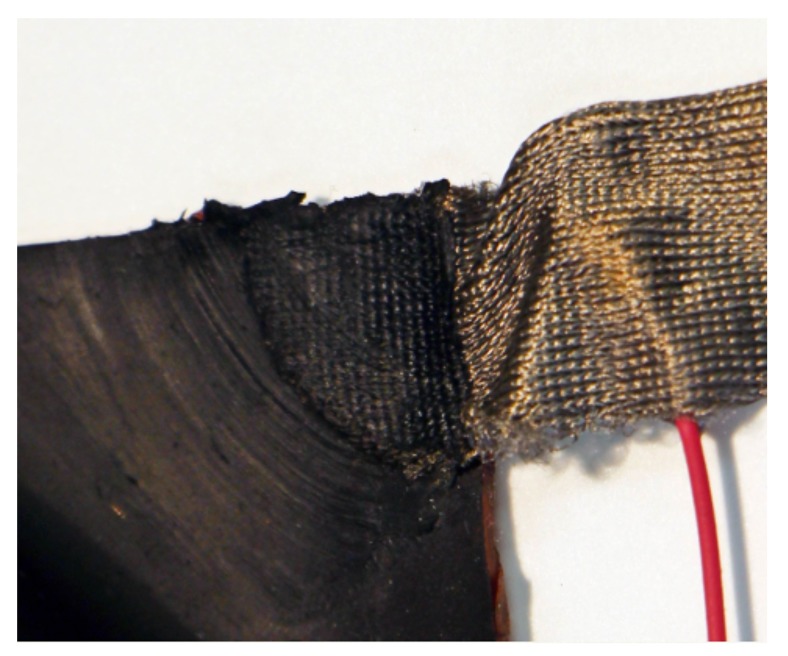
A close-up of CNT PDMS conductive cloth contact detail.

In each of the four corners there is an electrode. The electrodes are a quarter-circle cut from the same silver conductive fabric with a 1 cm-radius from the upper layer. The fabric is infused with the CNT-PDMS resin material from the coating of the lower layer and heat-cured onto the bottom layer as shown in Figure 4. A single electrical wire is then soldered to each electrode to provide an electrically conductive connection. The contact resistance of the corner electrode is approximately 500 Ω, and the flexible and stretchable properties of the skin are maintained at the electrode locations.

The CNT-PDMS layer is used as a bulk resistive sheet for position at a given stretch level, and force estimation is derived from the contact resistance between the CNT-PDMS layer and the conductive fabric layer, through the holes in the perforated layer. The piezoresistive effect of CNT-PDMS composite is used to estimate the current stretch level of the skin; however, this has a time behavior with hysteresis, which was not studied here as it is assumed that the skin will not be rapidly stretching. If the skin is only to be stretched once, for example upon installation, and calibration is done in the stretched state, the piezoresistive effect of the CNT-PDMS material may be ignored.

**Figure 5:**
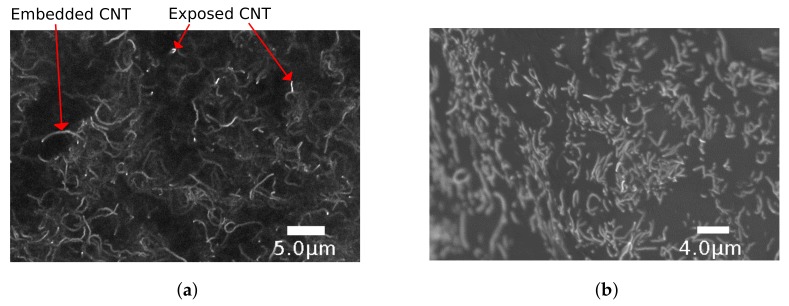
(**a**) SEM detail image of sheet surface region showing carbon nanotube matrix embedded within silicone and exposed CNT ends protruding from surface (15.0 kV × 10.0 k magnification in 5.1 mm SE(U) mode); (**b**) SEM detail image of nanotubes embedded within torn edge confirms significantly higher CNT density within the CNT-PDMS material than that expressed near the sheet surface (0.6 kV × 13.0 k magnification in 2 mm LA0(U) mode).

**Figure 6:**
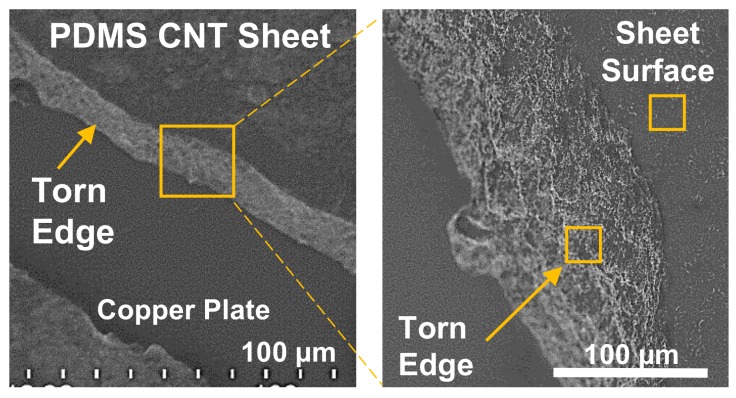
SEM overview of carbon nantoube-doped silicone skin sample (**left**) with a torn edge at 0.6 kV × 450 k magnification in 1.9 mm LM(UL) mode. The detail image (**right**) shows a close-up view of the torn edge with an apparent higher concentration of nanotube ends visible there than at the sheet surface at 0.6 kV × 3.50 k magnification in 2.0 mm LA0(U) mode; the yellow boxes indicate the approximate region shown in the detail images immediately below.

The CNT-PDMS was imaged with an SU8230 ultra-high resolution cold field emission Scanning Electron Microscope (SEM) (Hitachi Ltd., Tokyo, Japan) to evaluate the consistency and protrusion of the CNTs within the CNT-PDMS sheet. A small sample was prepared for the SEM that exhibited a mechanically torn-off and sheared-off region to investigate both cross-sectional and surface distributions. No observable differences were noted between cross-sectional regions obtained by tearing (shown, Figure 5) or shearing (not shown). An overview image was first taken of the transition region between the surface and cross-sectional edge achieved via tearing, Figure 6 (left), and a close-up of the edge (right). A typical region of interest on both the torn edge and sheet surface was identified for enhanced magnification to detail the CNT behavior at the sheet surface (Figure 5a) and within the edge (Figure 5b). Note that the higher voltage used in Figure 5a should provide more penetration further beyond the surface boundary to reveal deeper structures. From Figure 5a, it appears that exposed CNT fibers occur within a typical spacing of roughly 10 μm. The fact that the CNT protrudes in a hairlike fashion from the surface allows the contact resistance to vary sufficiently for forces estimation, as shown later.

To allow repeatable tests of human interaction with the skin while recording forces, a synthetic index finger was created as a replica of the index finger of an adult male. This synthetic finger was cast out of silicone rubber with A35 durometer (PlatSil 71-35, Polytek Development, Easton, PA, USA). To provide more realistic rigidity of a skeletal structure and allow a fixture point for a load cell, a wooden dowel of 6.5 mm diameter was placed in the mold of the finger.

### 2.2. Electronics and Algorithm Design

The electronics consist of four wires attached to each of the corner nodes and one wire connected to the the conductive cloth upper layer. These five wires were attached directly to the GPIO pins of a microcontroller (ATmega328, Atmel Corporation, San Jose, California). The GPIO pins were programmable to be set either as an analog input, ground, $V_{dd}$ or high impedance (set as an input, but not read).

**Figure 7:**
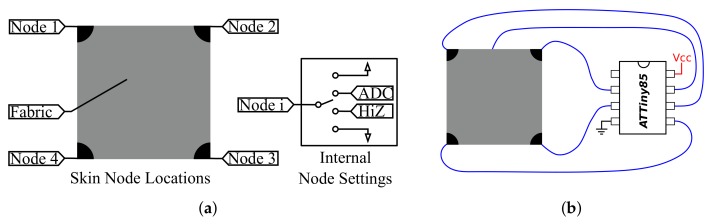
Smart skin electronic node schematic (**a**) and minimal smart skin setup requires supporting interface electronics between microcontroller and skin pad (**b**).

This allows $4^{5}$ permutations of which many are not useful (for example, all pins set to ground). To estimate the position of a touch, the permutations shown in Table 1 are used. This utilizes the top fabric node as the analog input, and applies $V_{dd}$ and ground to the four corners in different patterns to allow estimation of the position of the touch. For example, see the node values in Figure 7a and the top two rows in Table 1, which provide an estimate of the horizontal and vertical locations.

In order to estimate forces, an estimate of the contact resistance was used, where higher contact resistances correspond to lower forces. This is presumed to be due to the perforated nature of the intermediate layer, where increased force causes additional connections to be made through more of the holes, increasing the area of contact. The contact resistance was measured by calculating the ratio of the contact resistance to the bulk resistance of the pad. Since the bulk resistance of the pad should stay constant for different interaction forces, this allows a measurement of the contact resistance and therefore the force. The fabric node was set to either ground or $V_{dd}$ and at least one corner node was set to the opposite, either $V_{dd}$ or ground. Then a different corner node was used as an analog-to-digital converter to read the contact resistance ratio. This yields up to 16 different permutations with eight each being the opposite of the other eight that could be used to estimate force. The eight used are shown in Table 2.

The permutations outlined in Table 1 and Table 2 require the pins to be set differently so that each permutation used must be set up and read in sequence. The ability of pins to change between the four states used here is inherent to many microcontrollers, so additional circuitry is not needed. This allows a minimum setup that can be very small: for example, if an ATTiny85 (Atmel Corporation, San Jose, California) were to be utilized, the setup shown in Figure 7b would be sufficient, with only five wires and one microprocessor. Because the pins are set electronically, the process of setting the pins and reading the value takes approximately 1 ms on the hardware used here, so a tradeoff must be made between reading many permutations and receiving a fast update rate.

As the system was found to be accurately described by a third order polynomial in Section 2.3, it was assumed that the inverse mapping, where the voltages measured were used to predict the position, was also assumed to be third order. Therefore a two-by-three order polynomial with cross-terms was used to evaluate the position of the touch. The third order direction was in the direction that the voltage sweep went along: for example, the $V_{horiz}$ was used as the third order term when calculating *X*. An example of the fit used can be seen in Equation (1).

(1) $$\begin{array}{c} X=\left[\begin{array}{cccccccc} V_{h}^{3} & V_{h}^{2}V_{v} & V_{h}V_{v}^{2} & V_{h}^{2} & V_{v}^{2} & V_{h} & V_{v} & 1 \end{array}\right]\cdot \\ {\left[\begin{array}{cccccccc} a_{7} & a_{6} & a_{5} & a_{4} & a_{3} & a_{2} & a_{1} & a_{0} \end{array}\right]}^{T} \end{array}$$

The calibration data was used to create coefficients utilizing this equation, and those coefficients were programmed on the microcontroller, enabling the acquisition of real-time position data. This data was then leveraged by the robot controller in the collaborative interaction experiment.

The relation between node voltages and applied force was not found to have parametric model. Instead, we utilized a Neural Network (NN) regression to relate the node voltage to the applied force. For this approach we used the eight permutations found in Table 2. Using a known applied force, we then fit the NN regression weights in a three-layer NN setup. Hidden layer 1 consisted of ten nodes, hidden layer 2 consisted of five nodes, and the output consisted of one node. The two hidden layers utilized a sigmoid activation function while the output node used a pure linear activation function. The calibration data for force was collected and used in a standard back-propagation scheme to train the model. The feedforward model was then implemented in the microcontroller.

In many robotic cases, estimation of continuous force values is not necessary. Instead, it is often preferable to provide alerts when force values exceed a threshold or assume certain discrete force levels. Here, the force estimation from the NN regression was used to estimate three discrete force levels: Low Force (2.5 N < *F* < 7.5 N), High Force (12.5 N < *F* < 17.5 N), and No Force (*F* < 0.5 N).

For the most basic force use, any force information above a certain threshold is assumed to be contact. This ensures that the signal could be sent directly to the emergency stop circuit of the robot if contact is to be avoided, or could validate that position data is corresponding to an actual touch.

### 2.3. Finite Element Modeling and Simulation

**Figure 8:**
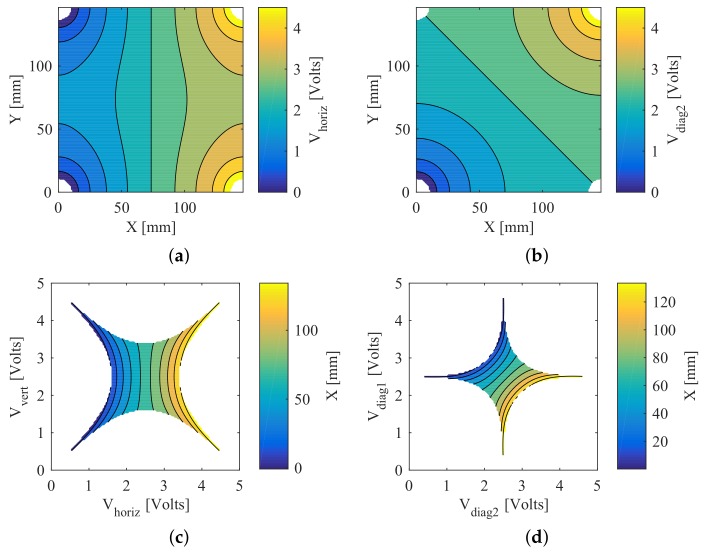
Finite element simulation with (**a**) linear and (**b**) diagonal sweep. Inverse mapping of (**c**) linear and (**d**) diagonal voltages to position (white area does not map to workspace, i.e., it indicates voltage combinations that were never observed).

The position-sensing algorithm was based on a finite element model of conduction diffusion. The methods outlined in [34] were modified to include a 14.7 cm square with external Neumann boundaries and four pads in each corner that were quarter circles of 1 cm radius with Dirichlet boundaries. This was numerically solved using MATLAB’s (MathWorks, Natick, MA, USA) Partial Differential Equation toolbox solving an n=10,417 node steady-state diffused conduction system: −∇·(σ∇(V))=q,E=∇(V), with V=5 volts. The results of the simulated diffusion can be seen in Figure 8a, showing one of the linear permutations of corner electrode voltages, and in Figure 8b for a single diagonal permutation.

A two-dimensional polynomial fit was used to predict the voltage mapping. This polynomial was fit to the data using least squares regression with bisquare robustness correction. This allows a microprocessor to evaluate in real-time due to the minimum number of floating point operations needed. The simulated voltages display even and odd symmetry, which is what led us to use quadratic and cubic terms. As these terms were the minimum required to account for the curvature observed in Figure 8a,b, the order of the polynomial model mapping voltage to position was set to the same degree.

While a fit of the predicted voltage at a given point, as [34] showed, is useful, for the skin application the inverse solution was required so that a position estimate could be given for a given observed voltage. This was achieved by simply fitting the X and Y dimensions each to a third-order polynomial of the voltages $V_{1}$ and $V_{2}$, which are explained further in Table 1. The fitting of the polynomial for X is shown in Figure 8c, where the white area represents voltage combinations not present in the simulation.

**Figure 9:**
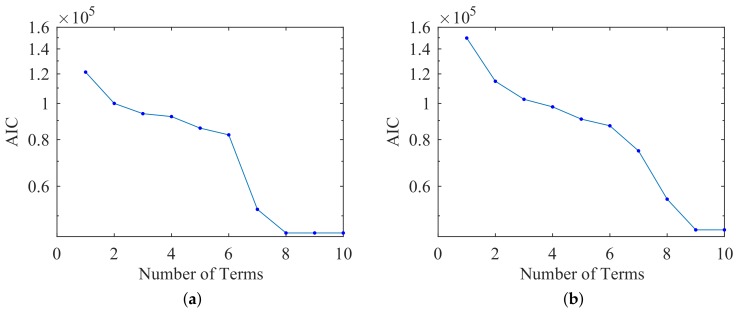
Akaike information criterion (AIC) of best inverse mapping polynomial fit to (**a**) linear and (**b**) diagonal FEM.

To evaluate the level of overfitting, we ran an Akaike Information Criterion (AIC) analysis. This determined which terms from the full third-order polynomial with cross-terms were required and which were extraneous (Equation (2)). We used the residual sum of squares (RSS) to evaluate a model’s maximum likelihood estimate. Figure 9a shows the diminishing returns beyond eight terms, corresponding to a third-order by second-order polynomial with cross-terms.

(2) $$AIC=2k+n\;ln(\frac{RSS}{n})$$

In addition to the linear sweep method from [34], we found that a diagonal sweep, holding the other two corners at high impedance, provided a similar level of quality to the linear method with the same polynomial order.

We again evaluated the level of overfitting by running an AIC analysis to determine which terms from the polynomial were required and which were extraneous. Figure 9b shows the diminishing returns beyond nine terms, corresponding to a third-order by second-order polynomial with cross-terms.

These two methods can also be combined into a single least-squares fit, which provides a very small benefit in simulation but can be used in the physical system to reduce Gaussian error by using four separate readings instead of just two.

### 2.4. Experimental Design

The proposed smart skin was evaluated through a series of experiments to validate our inverse kinematic model and to assess utility with regards to collaborative robotics. A variety of calibration routines were required to validate this inverse model. The first calibration routine was performed for the two-dimensional position of force application. The second calibration routine fit the parameters for the force magnitude. The third calibration routine validated the inverse kinematic model with the skin in multiple stretched states.

#### 2.4.1. Experiment 1

**Figure 10:**
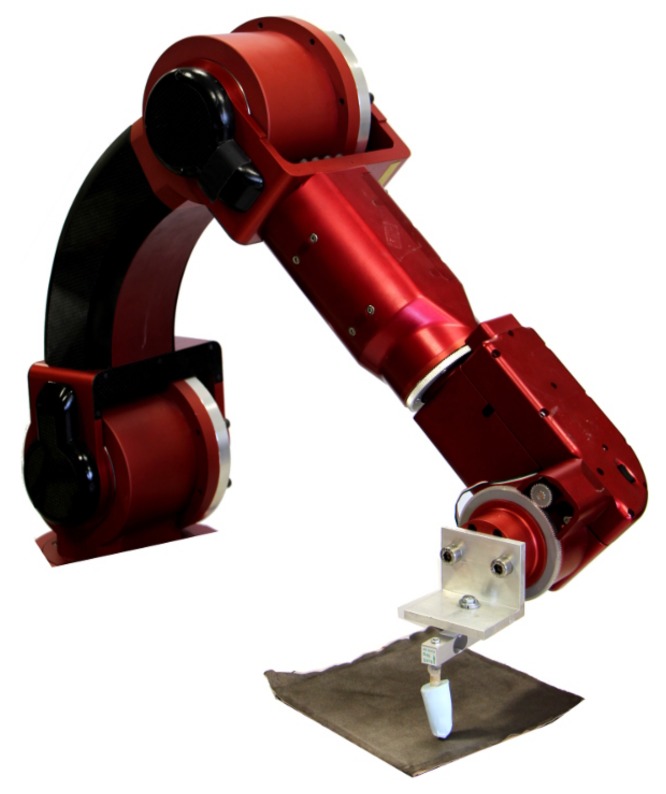
A custom robotic arm was used to calibrate position sensing in typical human tactile interactions by applying known forces in a known two-dimensional grid via a human finger replica.

The position-sensing of the skin was tested using a calibration routine involving a fixed force applied in a regular two-dimensional grid with 1 cm spacing. This regular grid was achieved by the use of the Complete Operating Room Robot for Virtually Unassisted Surgery (CORVUS) [37], a robot arm that allowed precise positioning of the touches. To provide accurate simulation of a human touch, a molded finger made of silicone was used as shown in Figure 10 and outlined in Section 2.1. The force was continuously monitored through the use of a load cell, and the robot applied a constant force through the use of a controlled compression distance. These X−Y positions were combined with the voltages measured for each of the permutations in Table 1. This data was then used in MATLAB to find the coefficients for the third-order polynomial as outlined in Section 2.2.

#### 2.4.2. Experiment 2

The calibration of force was achieved through the same synthetic finger and force-detecting load cell as in Experiment 1. Both the measured force and the voltage permutations from Table 2 were recorded simultaneously at 30 Hz throughout the touch event. The force data was collected at a single location in the pad’s center.

This data was then used to train the Neural Network regression model, where the input variables consisted of the voltage permutations from Table 2 and detailed in Section 2.2.

#### 2.4.3. Experiment 3

**Figure 11:**
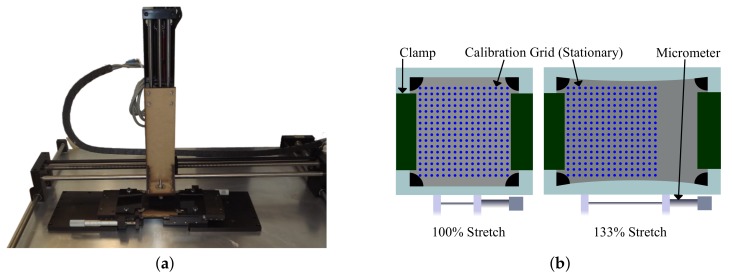
Stretch evaluation setup is shown in (**a**) while a schematic of the stretch evaluation setup is shown in (**b**).

To evaluate accuracy in a stretched state, the skin was placed under an XYZ linear stage shown in Figure 11a that automatically actuated forces in a regular grid. The skin sample was placed in a micrometer stage on a stationary table. On the *Z* stage, a conductive end-effector followed a downward trajectory onto the skin. The skin was stretched by bracing the left and right 5 mm strips in a vice clamp attached to a micrometer. The calibration grid was set to a constant 5 mm spacing in *X* and *Y* for each stretch state shown in Figure 11b. The grid location and sensor data for each touch was logged for one second with an average taken for each node permutation. The skin was tested at a neutral 100% stretch state (90 mm between clamps), a 106% stretch state (95 mm between clamps), a 111% stretch state (100 mm between clamps), a 122% stretch state (110 mm between clamps), and a 133% stretch state (120 mm between clamps). The skin was tested without a top fabric layer in order to avoid extraneous contact artifacts between clamped layers. Instead, the force-actuating end effector on the *Z* stage was covered with the conductive fabric node. The true position and conductance data was then fit to the proposed polynomial surface model for each stretch level to assess the degree to which the polynomial fit method works across a range of stretch levels.

Additionally, during the stretch test the bulk resistance of the pad was measured by adding a shunt resistor to each node and calculating the voltage differential across the resistor to estimate the current flow into or out of the pad. This sensing modality requires yet another permutation, where the fabric and two nodes are high impedance, with one node grounded and one node at $V_{dd}$. This was logged for each touch at each stretch level. This additional circuitry would only be necessary in practice if the skin was to be stretched or unstretched actively during use.

#### 2.4.4. Experiment 4

**Figure 12:**
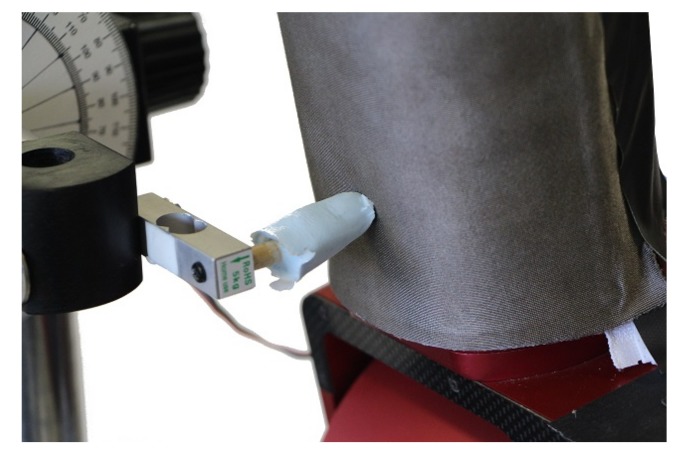
Known forces were applied to the skin mounted on a link via the silicone-cast finger attached to a load cell.

Multiple experiments were conducted to assess the utility of the smart skin in a collaborative robotic setting. The first experiment was designed to evaluate the skin’s use as an emergency stop detector on an industrial robot. The skin was applied to the surface of the distal link of a custom-made industrial robot (CORVUS). The robot’s emergency stop circuitry was then attached to the microcontroller of the smart skin via a solid-state relay. The skin was set to react to any non-zero force as an emergency stop event, and the microcontroller was sampling at 150 Hz. The CORVUS robot proceeded on a trajectory at 3 mm/s towards the synthetic finger, which was mounted on a load cell in order to measure the force that the robot applied to the finger as shown in Figure 12. In addition to the force data, the state of the emergency stop circuit was recorded for every timestep. To evaluate repeatability of the emergency stop functionality, the experiment was run 20 times.

**Figure 13:**
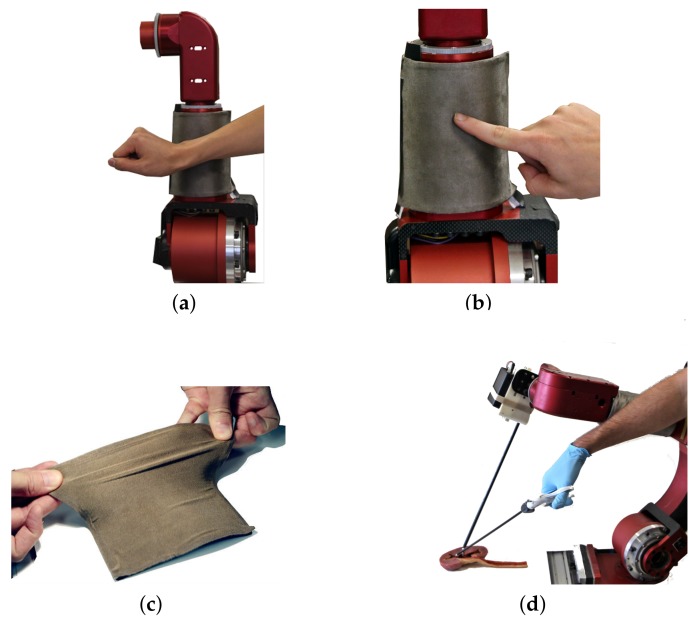
The skin is shown with: (**a**) An arm placed in the path of the robot; (**b**) A finger placed in the path of the robot; (**c**) Evidence of physical stretching near 150% and (**d**) An example collaborative use case.

In order to further evaluate the emergency stop functionality of the smart skin, the test was run with the synthetic finger replaced by a human test subject’s arm (Figure 13a). The skin was programmed to trigger an emergency stop in a similar manner to the above experiment, and again the robot was commanded to take an unsafe trajectory that intercepted the human. The experiment was repeated 20 times to test for repeatability.

#### 2.4.5. Experiment 5

The second functionality experiment was designed to evaluate the smart skin’s use in a human-robot interaction. The skin was placed on the CORVUS robot in the same manner as in Experiment 4; however, in this case the skin was programmed to detect the location of a touch and to forward that information to the robot, which was programmed to move in the opposite direction at a speed proportional to the estimated force. The direction was assumed to be from the point of contact towards the central axis of the robot link, which essentially made the direction anti-normal to the skin surface so that the robot would move directly away from the finger. The finger used was the same load-cell mounted synthetic finger as the above experiments, allowing the force to be monitored continuously during the interaction. The synthetic finger was moved manually towards the skin and the robot was either programmed to remain still (control) or take evasive action.

## 3. Results

The skin used in the experiments met the requirements of stretchability and flexibility, with Figure 13c showing stretch to approximately 150% of rest size without tearing. The skin also proved resilient to the repeated application of stress, exhibiting no change in electrical properties.

The application of the skin attached to the CORVUS arm’s irregular surface was successful, with the skin still functioning as expected, and is shown in Figure 13b.

An overall outline of the results concerning model validation and collaborative experiments can be found in Table 3.

### 3.1. Experiment 1

**Figure 14:**
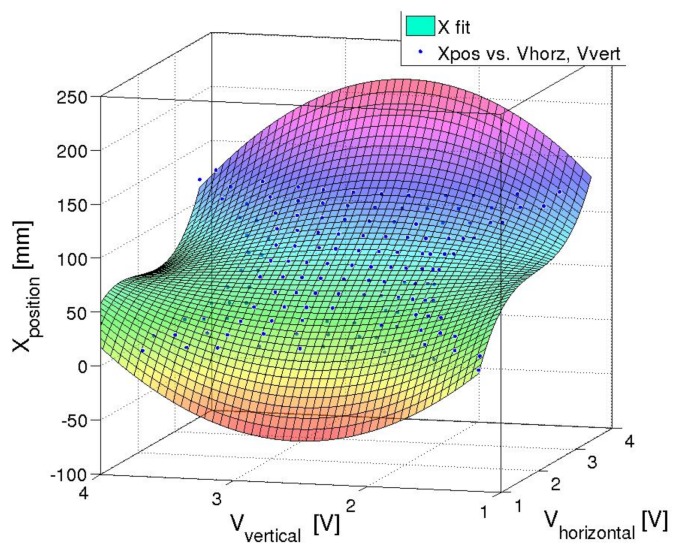
Polynomial fit in X $\left(R^{2}=0.993\right)$.

**Figure 15:**
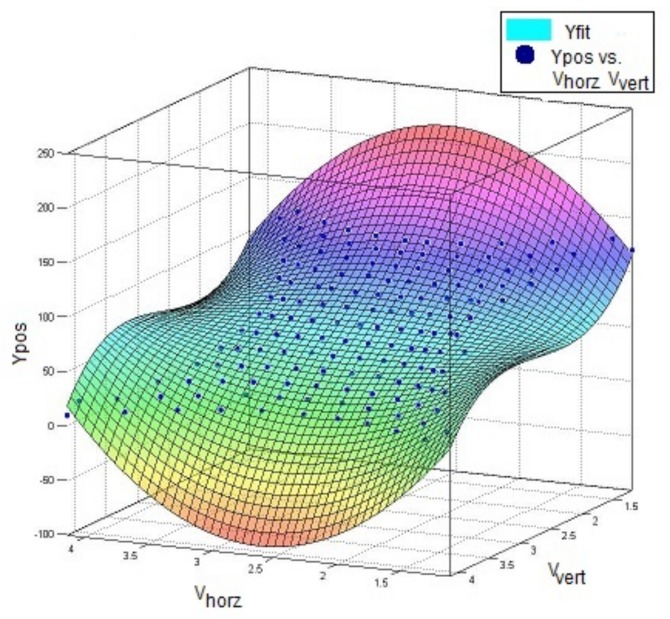
Polynomial fit in Y $\left(R^{2}=0.998\right)$.

The calibration of the skin’s position estimation was used to determine the polynomial coefficients with $R^{2}$ values of 0.9934 for the fit in the *X* direction and 0.9978 for the fit in the *Y* direction. The position accuracy had a mean absolute error of 3.32 mm and the root mean square error was 7.02 mm. The corners and boundaries showed the highest error rates, and 90% of all position error was below 5.7 mm. Figure 14 shows the three-dimensional polynomial fit of the *X* direction, and Figure 15 shows the three-dimensional polynomial fit of the *X* direction.

### 3.2. Experiment 2

**Figure 16:**
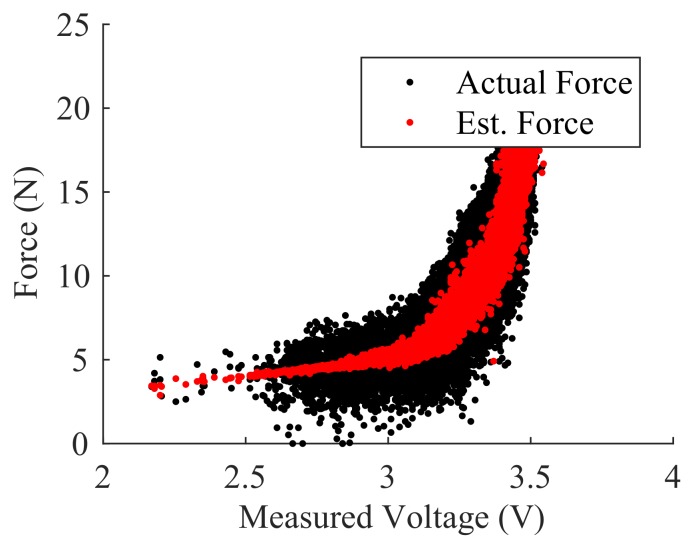
Neural Network model relating node voltage to force $\left(R^{2}=0.875\right)$.

The results of 144 different touch incidents at the same central location of the skin are plotted in Figure 16. A Neural Network (NN) regression model relating node voltage to true load-cell force showed an $R^{2}$ value of 0.875.

**Figure 17:**
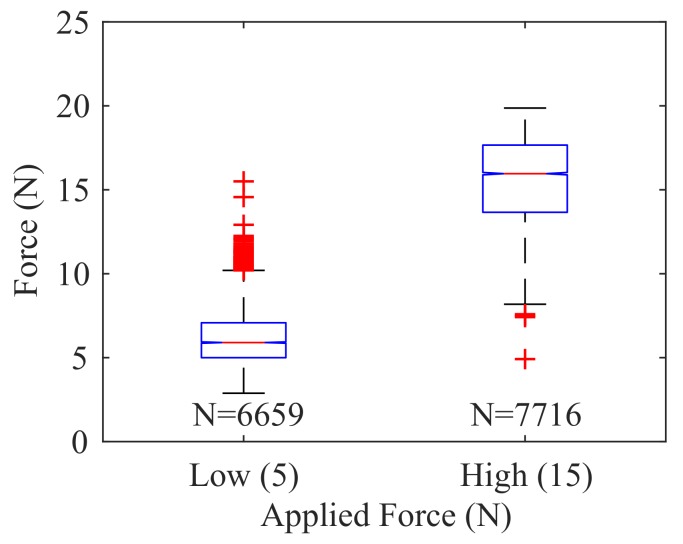
Estimated force versus force magnitude category.

Additionally, a force magnitude classification was performed using the NN regression wherein the true force was used to segment the data into two categories: Low Force (2.5 N < *F* < 7.5 N) and High Force (12.5 N < *F* < 17.5 N). Using the voltage data from these two categories, the output of the NN regression was plotted against the two ranges of force magnitude (Figure 17). The results indicate clear separation between the low and high force ranges.

### 3.3. Experiment 3

**Figure 18:**
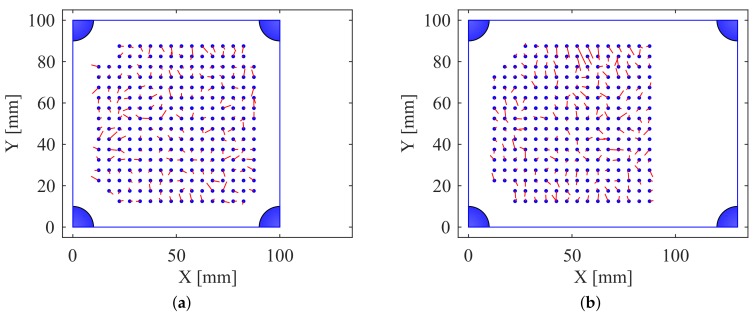
The skin (**a**) unstretched and (**b**) at 133% stretch, with the actual point pressed represented by blue circles and the red line signaling the point estimated by the inverse mapping polynomials to indicate error.

The stretched skin provided good polynomial fits in *X* and *Y* (Figure 18), with no systemic differences between the unstretched and 133% stretch cases.

**Figure 19:**
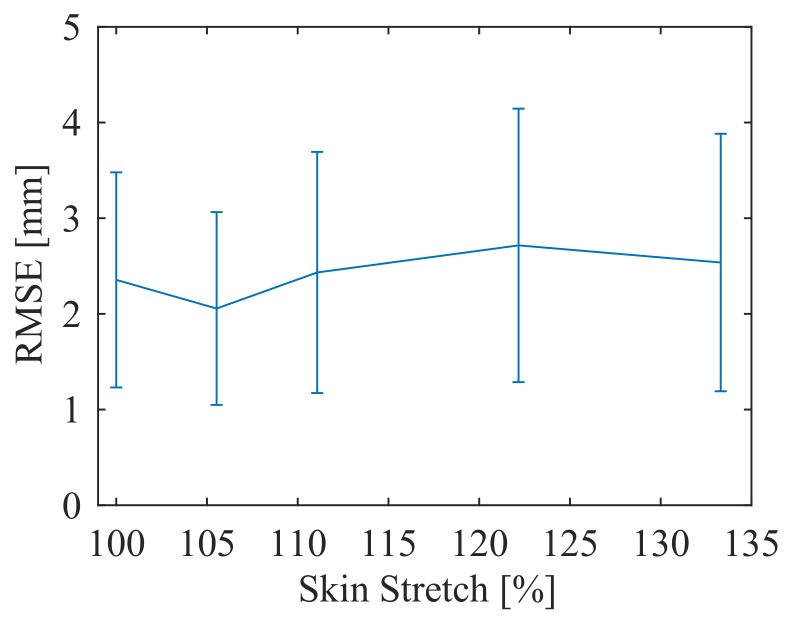
Accuracy of position estimation over various stretch levels. Error bars show (+/−) one standard deviation.

The results of the stretch test show that the error does not increase as the skin is stretched (Figure 19), staying at approximately 2.5 mm with a standard deviation of 1.5 mm. This allows the skin to be calibrated at any number of stretch levels.

**Figure 20:**
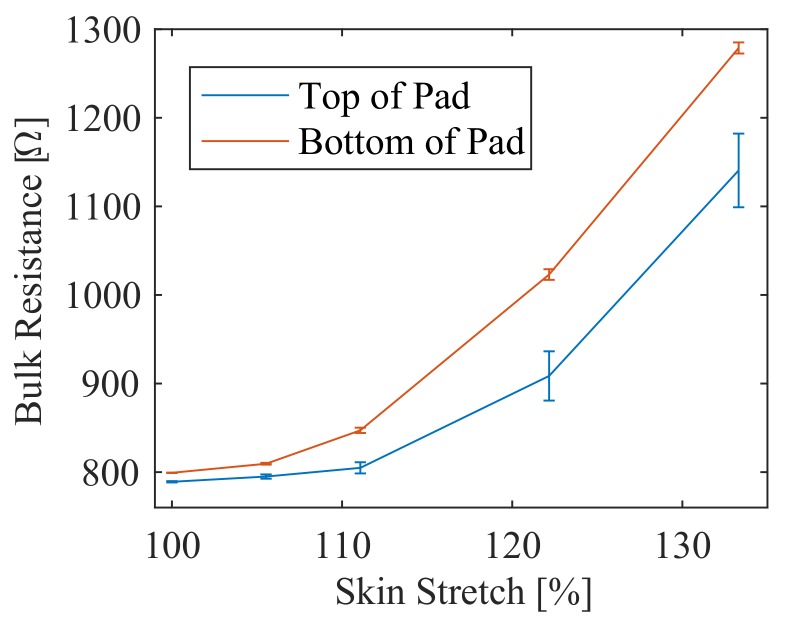
Bulk resistance of the pad between electrode pairs along the top and bottom of the pad for various stretch levels. Error bars show (+/−) one standard deviation.

The results of the stretch test also show that the bulk resistance of the pad (as measured between electrode pairs along the top and bottom of the pad using shunt resistor current) varies with stretch (Figure 20). Each stretch level is more than two standard deviations from the next, allowing estimation of stretch level online by measuring the current. A calibration for that stretch level could then be loaded to provide adaptable stretch functionality.

### 3.4. Experiment 4

**Figure 21:**
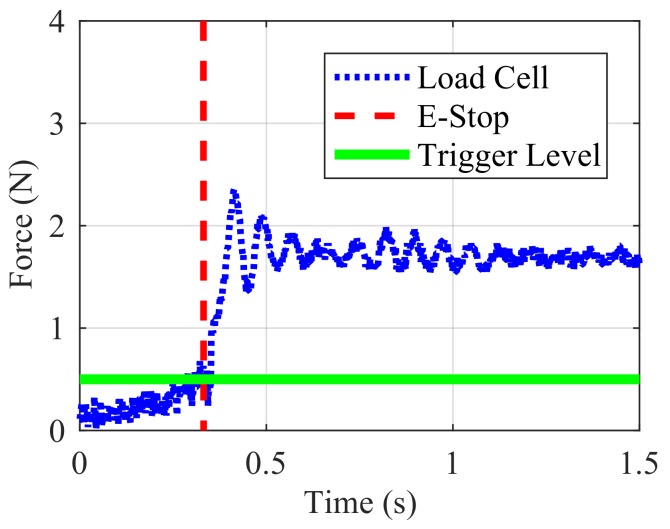
Force on finger with emergency stop.

The results of the finger touch triggering an emergency stop can be seen in Figure 21. The horizontal green line demarcates a force that the skin will register as a touch, which was 0.5 N. The vertical line shows the time at which the skin detected a touch and sent an emergency stop command to the robot. The robot took approximately 100 milliseconds to come to a stop, and oscillations continue as the robot’s entire mass dissipates the kinetic energy. The 20 iterations that were run all resulted in successful detection of touch and triggering of the emergency stop of the robot.

In the second emergency stop experiment involving a human subject, the interaction force was not measured quantitatively; however, no significant force on or movement of the human arm was observed or reported during the tests. The 20 iterations that were run all resulted in successful detection of the touch and triggering of the robot’s emergency stop function.

### 3.5. Experiment 5

**Figure 22:**
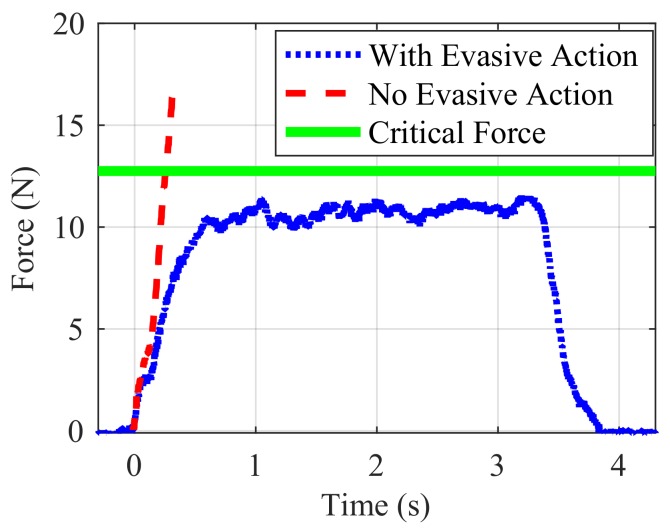
Force on finger with evasive action.

The results of the collaborative control experiment can be seen in Figure 22. The version of the experiment where the robot was not programmed to take evasive action (control) shows a rapidly increasing force to the limit of the load cell, without decreasing due to the fact that the target is never reached. The collaborative control version of the experiment, however, shows that the interaction force remains below the example critical force of 12.5 N, staying at approximately 11 N until the finger reaches the target position and stops moving.

## 4. Discussion

The *X* and *Y* calibration provided an accuracy of below one centimeter, which is often sufficient for many bulk sensing applications, such as those performed by human skin. This would provide sufficient accuracy to allow situational awareness to a robot with a soft and durable continuous skin. A limitation of this specific experiment, however, is that multitouch was not tested, and robust multitouch does not seem possible with the specific methods described here. Future work should investigate using node currents as well as node voltages to detect that a multitouch is occurring and possibly estimate the multiple touch locations.

The force measurement provided the ability to differentiate between low and high forces, but discrete force estimates do not provide enough repeatability to allow continuous force measurements. The continuous force estimate had an $R^{2}$ value below 0.9 and therefore may not provide sufficient accuracy for some applications. A limitation of the experiment is that the force measurement was performed with a synthetic finger (effective area on the order of 1 cm${}^{2}$), and due to the contact-resistance estimation method the force estimate is highly dependent upon the geometry and hardness of the contacting object. The soft human-like object used here was chosen in order to provide the most similarity to a real-world human-robot interaction. Also, the softer object was considered the more important object to detect from a safety perspective, and is similarly used in the emergency stop experiment. Additionally, the experiment was only performed at one location and one stretch level. Therefore, future work should focus on further characterizing the force estimation, especially contacting objects specific to the desired use case or a series of objects with defined sharp and a known stiffness, for instance, a series of plastic cylinders with known surface area and Young’s modulus.

The stretch calibration showed that accuracy does not diminish as the skin is stretched, although the change in calibration for each stretch level did not seem to follow an expected pattern. Therefore, a separate calibration should be done for each stretch level expected for the desired use case. If the use case requires the skin to be in a rigid state, such as stretching and permanently affixing to a rigid robot link, the calibration should be done in place to provide maximum accuracy while automatically calibrating to a functional coordinate frame. Calibration can be completed with approximately 25 known locations in a relatively simple procedure. If the use case involves dynamic motion, such as stretching the skin around an elbow of a robotic joint, the skin should be calibrated to the full range of stretches expected, and the bulk resistance of the pad should be measured in real time to determine the stretch level and appropriate calibration. A limitation of this experiment is that the full skin was not tested and the fabric layer was substituted with a fabric-covered tip while the perforated layer was omitted. This could have provided better contact than is realized when the entire skin is used, which could explain why the position results for the stretch test outperformed those for the whole skin in Experiment 1. Further work should determine the effect of repeated stretch on functionality, compare the fabric-covered tip to the whole skin, and evaluate stretching modalities other than the uniaxial mode shown here.

The emergency stop experiment showed the usefulness of this sensor in a robotic system. The low-threshold force of just half a Newton is roughly equivalent to the weight of a tennis ball, and the test was performed with a human arm and finger without causing any discomfort. A limitation of this experiment was that it was performed in a high-speed mode, where the position calculations were not performed and only emergency stop decisions were made.

While the force experiment showed that discrete force estimations are quite noisy, the collaborative control experiment showed that the force could be used in a feedback loop to roughly maintain an interaction force. The quality of the interaction will be dependent both upon the robot used and the contacting object in a collaborative environment with humans. This could be acceptable as larger contact objects would correspond to faster reactions, which would likely be appropriate.

Qualitative analysis was performed on the SEM scans of the skin material. The roughly 10 μm spacing of carbon nanotubes protruding at the surface for viable contact places a theoretical lower bound on the positional resolution accuracy achievable with this method of doping PDMS with CNT. However, given the relatively low expression of CNTs near the surface of the sheet when compared to those within the bulk body as shown in Figure 5, it is likely that the effective electrical contact area of the nanotubes can be increased. It should also be noted that due to electrical constraints in practice (such as quantization in analog-to-digital conversion), the effective resolution is practically bounded and lower than what the CNT technology could potentially provide.

In order to enable advanced human-robot interactions in a safe and reliable manner, a robot needs the ability to sense contact not just at the end effector, but on any point on the body. This paper presented a stretchable, flexible skin sensor capable of discerning between discrete force values while providing a typical positional accuracy of 5.7 mm. The two-dimensional potentiometer method of position estimation shown here can provide two independent estimations of position for any rectangular skin shape while also providing a crude force estimation. The ratiometric nature of the skin surface allows continuous measurement and scaling up or down either dimension of the skin. Although skins were not tested far beyond the 10 cm size, theoretically the skin could be scaled from approximately 1 cm to 1 m utilizing the manufacturing methods outlined here.

A significant limitation of the skin as shown in this paper is that the outer surface is a conductive node, and therefore if it is contacted by anything electrically active the sensor could be damaged. Future work should address this by investigating the use of another PDMS sheet with CNT as the top layer, potentially with a different loading of CNT or a more conductive material such as silver nanowire doped PDMS [38], while leaving a waterproof PDMS layer exposed on the outside of the sensor. This top layer could also have four nodes, leading to a full eight nodes, with many more permutations available for features such as multitouch.

During the experimentation, repeated stretching of the sensor did not seem to influence the accuracy of the localization of touches, and the PDMS substrate that makes up the bottom layer is inherently durable and stretchable. The stiffness of the skin is heavily influenced by the thickness of the bottom layer, allowing different stretchability levels. The size of the holes in the middle mesh layer could be tuned to allow different threshold forces, which would be beneficial if using the skin as an emergency stop sensor as in Experiment 4. The layered functionality could also allow for multiple sets of the layers outlined in this paper to be sandwiched together. This could allow different triggering forces for different layers, or provide redundant readings for increased accuracy and safety.

This skin allows a lighter force threshold than commercial robots with torque sensors or estimators in their joints, such as the UR5 or KUKA robots. The skin could work in concert with these robots’ sensors to increase the safe operating range or provide additional redundant force sensing for safe human robot collaboration in close proximity.

This paper outlined two possible implementations where the skin could be used either as an emergency stop detection sensor or to enable collaborative use in an interactive environment. However, the skin presented here is not limited to industrial robotics. It could also be used in other fields such as medicine, where the highly constrained surgical environment could benefit from robots having an increased awareness of their surroundings as shown in Figure 13d. Another medical application is prosthetics, which lack a skin-like touchability [11]. Localized touch sensations could be transmitted from this skin through a neural interface method such as in [39], but with the benefit of reduced cost compared to current sensors in use.

The skin outlined here provides a sense of touch to robots with an electrically simple design through the use of affordable but durable materials that allow stretching and flexing without losing the ability to provide sub-centimeter positional accuracy and an estimate of the force range.

## Figures and Tables

**Table 1:** Permutations used for position. Top four rows use linear method, bottom four rows use diagonal method.

Node #	1	2	3	4	Fabric
$V_{vert}$	Gnd	Gnd	$V_{dd}$	$V_{dd}$	ADC
$V_{horiz}$	Gnd	$V_{dd}$	$V_{dd}$	Gnd	ADC
$V_{dd}-V_{vert}$	$V_{dd}$	$V_{dd}$	Gnd	Gnd	ADC
$V_{dd}-V_{horiz}$	$V_{dd}$	Gnd	Gnd	$V_{dd}$	ADC
$V_{diag1}$	Gnd	HiZ	$V_{dd}$	HiZ	ADC
$V_{diag2}$	HiZ	Gnd	HiZ	$V_{dd}$	ADC
$V_{dd}-V_{diag1}$	$V_{dd}$	HiZ	Gnd	HiZ	ADC
$V_{dd}-V_{diag2}$	HiZ	$V_{dd}$	HiZ	Gnd	ADC

**Table 2:** Permutations used for force.

Node #	1	2	3	4	Fabric
$V_{f1}$	Gnd	Gnd	ADC	HiZ	$V_{dd}$
$V_{f2}$	Gnd	Gnd	HiZ	ADC	$V_{dd}$
$V_{f3}$	ADC	HiZ	Gnd	Gnd	$V_{dd}$
$V_{f4}$	HiZ	ADC	Gnd	Gnd	$V_{dd}$
$V_{dd}-V_{f1}$	$V_{dd}$	$V_{dd}$	ADC	HiZ	Gnd
$V_{dd}-V_{f2}$	$V_{dd}$	$V_{dd}$	HiZ	ADC	Gnd
$V_{dd}-V_{f3}$	ADC	HiZ	$V_{dd}$	$V_{dd}$	Gnd
$V_{dd}-V_{f4}$	HiZ	ADC	$V_{dd}$	$V_{dd}$	Gnd

**Table 3:** Summary Overview of Experimental Results.

	Experiment	Primary Result
1	Stationary Calibration	RMSE =7.02 mm
2	Force Calibration	$R^{2}=0.875$
3	Stretch Calibration	RMSE =2.5±1.5 mm
4	Emergency Stop	Threshold =0.5 N
5	Collaborative Control	Collaboration Force =10 N

## Abbreviations

The following abbreviations are used in this manuscript:

MDPI	Multidisciplinary Digital Publishing Institute
DOAJ	Directory of Open Access Journals
PDMS	Polydimethylsiloxane
CNT	Carbon NanoTube
CNT-PDMS	Carbon Nanotube Doped Polydimethylsiloxane
CORVUS	Complete Operating Room Robot for Virtually Unassisted Surgery
SEM	Scanning Electron Microscope
NN	Neural Network
